# Decadal Trends in the Prevalence of Metabolic Syndrome in Economically Developed Regions in China

**DOI:** 10.1210/jendso/bvae128

**Published:** 2024-07-03

**Authors:** Tianxing Feng, Jiali Zheng, Xiaoxiao Wang, Yilei Wang, Ping Shen, Beili Zhu, Huiyan Zhao, Li Zhao, Yaqing Xu

**Affiliations:** Department of Medical Affairs, Shanghai Clinical Research and Trial Center, Shanghai 201203, China; Department of Epidemiology and Biostatistics, School of Public Health, Shanghai Jiao Tong University School of Medicine, Shanghai 200025, China; Center for RNA Therapeutics; Houston Methodist Research Institute, Houston, TX 77030, USA; Division of Health Check-up, Rici Health Care Holdings Limited, Shanghai 200232, China; Division of Health Check-up, Rici Health Care Holdings Limited, Shanghai 200232, China; Division of Health Check-up, Rici Health Care Holdings Limited, Shanghai 200232, China; Division of Health Check-up, Rici Health Care Holdings Limited, Shanghai 200232, China; Division of Health Check-up, Rici Health Care Holdings Limited, Shanghai 200232, China; Department of Epidemiology and Biostatistics, School of Public Health, Shanghai Jiao Tong University School of Medicine, Shanghai 200025, China

**Keywords:** metabolic syndrome, prevalence trend, Chinese, health check-up, gross domestic product per capita

## Abstract

**Objective:**

To estimate decadal trends in the prevalence of metabolic syndrome (MetS) in economically developed regions in China and its association with city economic levels.

**Methods:**

Using a comprehensive Chinese healthcare database, repeated cross-sectional studies were conducted on adults who had annual health check-ups from 2012 to 2021 in 4 economically developed cities. MetS was defined by the criteria of the Chinese Diabetes Society in 2013. The crude prevalence of MetS adjusted for sex and age was reported. The association between prevalence, calendar year, and city gross domestic product (GDP) per capita was analyzed by regression model.

**Results:**

158 274 participants aged 18 years and older were included. The unadjusted prevalence of MetS increased from 15.5% (95% CI: 14.2%-16.8%) to 20.0% (95% CI: 19.5%-20.5%) from 2012 to 2021. The adjusted overall prevalence has increased steadily from 12.8% to 20.8% after controlling age and sex (*P* < .001). Male and older age groups had a higher MetS prevalence. In the regression model of the association between the MetS prevalence, calendar year, and city GDP per capita, calendar year had a positive association with the prevalence (*P* < .001, 95% CI: 0.648-1.954) and city GDP per capita had a negative association (*P* = .030, 95% CI: −0.136 to −0.007).

**Conclusion:**

The MetS prevalence increased steadily in the economically developed regions in China among the health check-up population during 2012-2021. The MetS prevalence is shown to be negatively associated with GDP per capita in the study population.

The metabolic syndrome (MetS) is a pathologic condition characterized by abdominal obesity, dyslipidemia, hyperglycemia, and hypertension, which may substantially increase risk for cardiovascular diseases and type 2 diabetes mellitus (T2DM) [1]. The prevalence studies often used different definitions of MetS, making it hard to compare the population-level prevalence across countries or between studies in different time periods [2]. Trends of prevalence of MetS among US adults were increased from 2011-2012 to 2015-2016, not meeting statistical significance, but there was a significant increase observed among young adults aged 20 to 39 years (from 16.2% to 21.3%; *P* = .02 for trend) [3]. With the fast spread of Western lifestyles across the globe, MetS has increased substantially in some developing countries, especially among those most transformed toward the Western lifestyle, which has been closely related to MetS [4]. A few meta-analyses reported an increasing trend in prevalence of MetS in Brazil, China, Iran, Mexico, Vietnam, and Middle East counties [5-10]. In the meta-analysis study based on Chinese data, the pooled prevalence of MetS in China was 23.8% [95% CI: 17.7%-29.9%] during 2000-2005, and it increased to 27.0% (95% CI: 22.2%-31.8%) during 2010-2015 [5], although this difference between the two time periods was not statistically significant. However, the meta-analysis was limited by inconsistent diagnostic criteria as well as demographic and geographical characteristics of the study population. For instance, in the meta-analysis of MetS in mainland China [5], MetS diagnostic criteria proposed in 2005 by the International Diabetes Federation was used; however, an updated and adjusted version of diagnostics for Chinese individuals was established by Chinese Diabetes Society (CDS) in 2013. Many studies included only reflected the annual prevalence of MetS in a single region or city. These limitations would affect the estimation of the trend in the prevalence of MetS in a certain country or region. Our literature search suggested that most updated prevalence trends covering a decade period or longer were lacking. In addition, the correlation between the prevalence of MetS and economic development level should not be ignored, because the economic development level often parallels the level of obesity and incidence of T2DM [2]. For instance, a study from India reported that states with higher gross domestic product (GDP) per capita had a higher prevalence of diabetes [11]. Similar results were observed in Gulf Cooperation Council countries where the prevalence of T2DM was found to be significantly associated with higher GDP [12]. But there is a paucity of data on correlation between the prevalence of MetS and GDP. Hence, to address the need for an in-depth understanding of MetS prevalence trends, we selected 4 representative cities of different levels in the urban economic development and with different scales in the Yangtze River Delta, which is one of the top metropolitan regions and economic hubs in China. A health check-up database of adult urban residents was used to conduct this cross-sectional study. We chose this population because there is less heterogeneity of the population in the recent 10 years. The study population consisted of individuals who were employed and relatively young. MetS serves as a screening approach, rather than a disease diagnosis, for the purpose of identifying a subgroup of high-risk individuals who would benefit from clinical and population-based approaches targeting their lifestyle habits. Furthermore, we evaluated the association between the prevalence of MetS and GDP per capita to address if higher economic development level would be a strong protective factor of MetS.

## Methods

### Study Population

This is a repeated cross-sectional study using a comprehensive Chinese health care database managed by Rici Health Care Holdings Limited. Rici is a private-owned company providing health check-up service through national chain clinics. We limited our study to participants aged 18 years or older with available data of physical and blood measurements. We included only participants in 4 cities (Shanghai, Suzhou, Nanjing, and Nantong), where the data were continuously collected through 2012 to 2021. These 4 cities have witnessed rapid economic growth in the past decade, and they represent different city levels of GDP per capita and urban population. The data used in this study were anonymized when exported from the Rici Healthcare database. Approval for this research was obtained by the ethical review committee of Rici Health Care. Informed consent of each subject was exempted because this study used only unlinkable, anonymized data for retrospective study.

### Definition of Metabolic Syndrome

The diagnosis of MetS mainly was as described in the 2013 CDS guideline [13]. MetS was defined when a person has 3 or more of the following components: (i) waist circumference of ≥ 85 centimeters (33.5 inches) in women and ≥ 90 centimeters (35.4 inches) in men; (ii) fasting plasma glucose ≥ 6.1 mmol/L (110 mg/dL), or glycated hemoglobin (HbA1c) ≥ 6.5%, and/or a self-reported history of diabetes; (iii) systolic blood pressure (SBP) ≥ 130 mmHg, or a diastolic blood pressure (DBP) ≥ 85 mmHg, and/or a self-reported history of hypertension; (iv) triglyceride levels of ≥ 1.7 mmol/L; (v) high-density lipoprotein cholesterol (HDL-C) of < 1.04 mmol/L. HbA1c was used in the diagnosis of diabetes mellitus as per the report of the World Health Organization (WHO) and as justification of the cutoff value for the Chinese population [14, 15].

### Demographic and Other Covariates

Rici Healthcare information system collected data on demographic characteristics of all participants, including sex, age, and resident city. We classified age groups as 18-39 years, 40-59 years, 60-79 years, and ≥ 80 years old at the time they had health check-ups. Past medical history of hypertension and diabetes was ascertained by a positive response to the question by trained physicians: “Has a doctor ever told you that you have hypertension or diabetes?” Height, weight, waist circumstance, and blood pressure were measured during the physical examination on site. Body mass index (BMI) was calculated as weight in kilograms divided by square of height in meters. Detection of fasting plasma glucose, HbA1c, triglycerides, and HDL-C were tested in a fasting state. To ensure consistency across all clinics in the Rici Healthcare group, blood samples were sent to central labs where comparable laboratory measurements were taken. All data were recorded in the electronic health record system.

### Statistical Analyses

The crude prevalence of MetS during 2012-2021 was reported in the study population, and the stratified prevalence was reported by sex, age, resident city, and number of components meeting diagnostic criteria. The 95% CIs were generated for crude prevalence. All analyses were performed using Stata/SE 16.1 (StataCorp).

In describing the adjusted prevalence over time, restricted cubic splines were used to model the trends. Three knots were at year 2014, 2017, and 2020 to allow flexibility as well as to demonstrate the prevalence trends. The trends were displayed with stratification by age group or sex. We used the fitted model and the observed values of each person to predict the risk and show the average risk for a given year and category. As the sample size is large enough for normal approximation, we used the two proportion Z-test to compare the adjusted overall prevalence between 2012 and 2021. The difference of overall prevalence between male and female individuals was analyzed using the Standard Wald's test from Stata's logistic regression analysis. Values for the spline fitting plot with standard errors (SEs) were derived using the “margins” command in Stata.

To explore the association between the prevalence trend and GDP per capita, multiple linear regression model was used. The prevalence for each year was adjusted by age and sex beforehand according to the 2021 distribution and was used as a dependent variable. The *P* values of the explanatory variables were calculated by Stata's linear regression Wald test.

## Results

### Decadal Trends in Crude Prevalence of MetS

**Figure 1.**
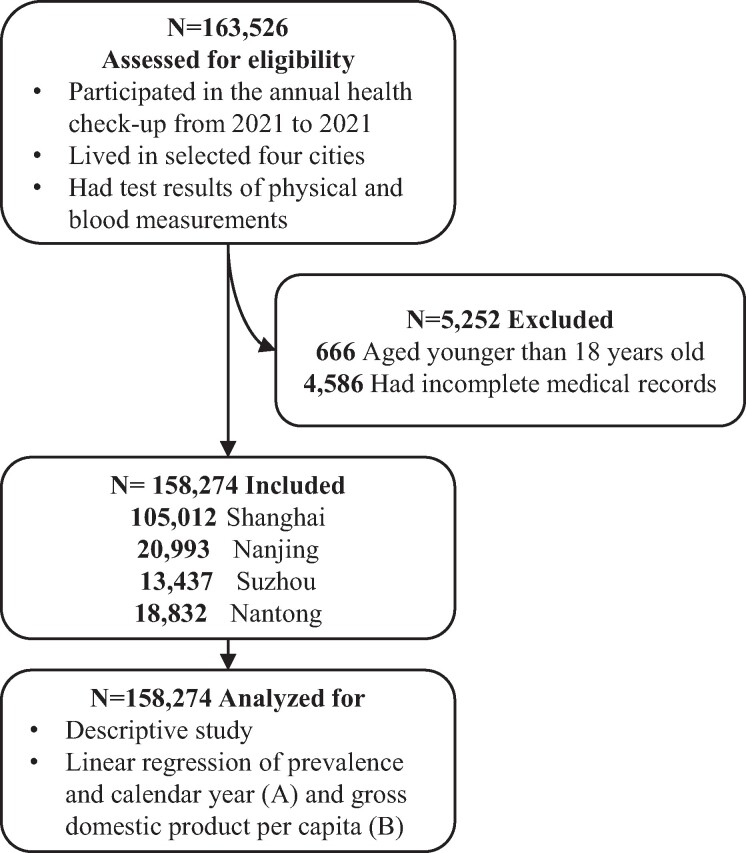
Flowchart of participant inclusion and analysis process.

A total of 158 274 participants aged 18 years and older with complete health check-up records were included from 2012 to 2021 (Fig. 1). Characteristics of the study population, by survey period, are shown in Table 1. In later calendar years, the proportion of men was higher. The mean age was slightly higher during 2016-2017 and had a larger fraction of the population aged 18-39 years. The crude prevalence of MetS increased from 15.5% (95% CI: 14.2%-16.8%) in 2012 to 20.0% (95% CI: 19.5%-20.5%) in 2021. For both men and women, the unadjusted prevalence of MetS was higher in 2021 than that in 2012. Unadjusted disease prevalence was considerably higher among men than women, from 22.3% (95% CI: 20.2%-24.4%) to 30.3 (95% CI: 29.6%-31.0%) in men and from 7.2% (95% CI: 5.8%-8.6%) to 8.8% (95% CI: 8.3%-9.3%) in women through 2012 to 2021. The increase in MetS prevalence over the most recent decade was generally observed in all age groups. When the study population was stratified by resident city, the prevalence of MetS among participants in Shanghai increased from 15.4% (95% CI: 14.0%-16.8%) in 2012 to 21.6% (95% CI: 21.0%-22.2%) in 2021 but slightly decreased in Nanjing, with a prevalence of 16.0% (95% CI: 7.7%-24.3%) in 2012 to 12.8% (95% CI: 11.7%-13.9%) in 2021. More results can be found in Table 2. Additionally, the number of MetS cases for different cities by sex with the number of participants of each city is presented in Table 3.

**Table 1.** Characteristics of the study population, 2012-2021

	2012 N = 2859	2013 N = 7724	2014 N = 9810	2015 N = 11 861	2016 N = 14 600
Men (%)	1564 (54.7)	4211 (54.5)	5359 (54.6)	6368 (53.7)	7852 (53.8)
Age (SD), years	39.3 (13.0)	39.4 (13.5)	39.8 (13.6)	40.2 (13.6)	47.6 (13.9)
18-39 (%)	1710 (59.8)	4576 (59.2)	5758 (58.7)	6775 (57.1)	4842 (33.2)
40-59 (%)	905 (31.7)	2411 (31.2)	3021 (30.8)	3782 (31.9)	6528 (44.7)
60-79 (%)	236 (8.3)	697 (9.0)	960 (9.8)	1210 (10.2)	3056 (20.9)
≥80 (%)	8 (0.3)	40 (0.5)	71 (0.7)	94 (0.8)	174 (1.2)
Body mass index (SD), kg/m^2^	23.4 (3.4)	23.5 (3.4)	23.6 (3.4)	23.5 (3.4)	24 (3.4)
Fasting blood glucose (SD), mmol/L	5.1 (1.0)	5.3 (1.0)	5.2 (1.1)	5.2 (1.0)	5.4 (1.2)
Hemoglobin A1C (SD), %	5.5 (1.6)	5.2 (1.5)	5.6 (1.7)	5.4 (1.9)	5.5 (0.9)
Systolic BP (SD), mmHg	119.1 (17.0)	120.3 (17.3)	120.5 (17.5)	120.4 (18.0)	124 (19.1)
Diastolic BP (SD), mmHg	74.2 (10.9)	74.7 (11.2)	74.6 (11)	73.8 (11.3)	74.9 (11.6)
Triglyceride (SD), mmol/L	1.5 (1.2)	1.5 (1.2)	1.3 (1.1)	1.3 (1.1)	1.5 (1.2)
HDL-C (SD), mmol/L	1.3 (0.3)	1.4 (0.3)	1.4 (0.3)	1.4 (0.3)	1.4 (0.3)

	2017 N = 17 524	2018 N = 18 352	2019 N = 22 363	2020 N = 23 694	2021 N = 29 487
Men (%)	9839 (56.1)	9416 (51.3)	11 511 (51.5)	12 299 (51.9)	15 272 (51.8)
Age (SD), years	47 (14.0)	41.1 (14.1)	41.2 (14.1)	41.7 (14.0)	40.9 (14.1)
18-39 (%)	6116 (34.9)	10 122 (55.2)	12 380 (55.4)	12 672 (53.5)	16 355 (55.5)
40-59 (%)	7681 (43.8)	5837 (31.8)	7041 (31.5)	8015 (33.8)	9887 (33.5)
60-79 (%)	3550 (20.3)	2226 (12.1)	2743 (12.3)	2759 (11.6)	3038 (10.3)
≥80 (%)	177 (1.0)	167 (0.9)	199 (0.9)	248 (1.0)	207 (0.7)
Body mass index (SD), kg/m²	24.1 (3.4)	23.7 (3.4)	23.8 (3.5)	23.8 (3.5)	24.0 (3.5)
Fasting blood glucose (SD), mmol/L	5.4 (1.2)	5.3 (1.1)	5.2 (1.1)	5.3 (1.1)	5.1 (1.1)
Hemoglobin A1C (SD), %	5.5 (0.8)	5.5 (2.3)	5.7 (2.4)	5.5 (2.4)	5.6 (2.8)
Systolic BP (SD), mmHg	124.1 (18.8)	122.7 (18.3)	123.7 (18.1)	123.3 (17.8)	123.2 (18.1)
Diastolic BP (SD), mmHg	75.0 (11.6)	73.8 (11.6)	74.6 (11.5)	74.8 (11.5)	74.8 (11.5)
Triglyceride (SD), mmol/L	1.5 (1.2)	1.5 (1.2)	1.5 (1.1)	1.5 (1.2)	1.5 (1.2)
HDL-C (SD), mmol/L	1.4 (0.3)	1.4 (0.3)	1.4 (0.3)	1.3 (0.4)	1.3 (0.3)

Abbreviations: BP, blood pressure; HDL-C, high-density lipoprotein cholesterol.

**Table 2.** Unadjusted prevalence of metabolic syndrome in the study population, 2012-2021*

	2012 N = 2859	2013 N = 7724	2014 N = 9810	2015 N = 11 861	2016 N = 14 600
Total population	15.5 (14.2, 16.8)	14.2 (13.4, 15.0)	11.4 (10.8, 12.0)	10.2 (9.7, 10.7)	15.3 (14.7, 15.9)
Sex					
Male	22.3 (20.2, 24.4)	20.9 (19.7, 22.1)	16.1 (15.1, 17.1)	14.5 (13.6, 15.4)	20.9 (20.0, 21.8)
Female	7.2 (5.8, 8.6)	6.1 (5.3, 6.9)	5.7 (5.0, 6.4)	5.1 (4.5, 5.7)	8.8 (8.1, 9.5)
Age, years					
18-39	14.7 (13.0, 16.4)	13.6 (12.6, 14.6)	11.0 (10.2, 11.8)	10.1 (9.4, 10.8)	7.7 (6.9, 8.5)
40-59	15.0 (12.7, 17.3)	15.6 (14.2, 17.0)	11.7 (10.6, 12.8)	10.4 (9.4, 11.4)	17.3 (16.4, 18.2)
60-79	22.0 (16.7, 27.3)	13.8 (11.2, 16.4)	13.0 (10.9, 15.1)	9.8 (8.1, 11.5)	22.4 (20.9, 23.9)
≥80	25.0 (0.0, 55.0)	5.0 (0.0, 11.8)	8.5 (2.0, 15.0)	13.8 (6.8, 20.8)	23.6 (17.3, 29.9)
City					
Shanghai	15.4 (14.0, 16.8)	15.4 (14.4, 16.4)	11.3 (10.5, 12.1)	9.0 (8.4, 9.6)	14.5 (13.8, 15.2)
Nanjing	16.0 (7.7, 24.3)	12.4 (10.8, 14.0)	11.0 (9.5, 12.5)	12.2 (10.8, 13.6)	15.9 (13.6, 18.2)
Suzhou	14.0 (8.2, 19.8)	11.3 (8.8, 13.8)	11.3 (9.0, 13.6)	9.5 (7.4, 11.6)	16.0 (13.9, 18.1)
Nantong	16.3 (11.5, 21.1)	13.2 (10.9, 15.5)	12.7 (10.7, 14.7)	13.3 (11.5, 15.1)	19.4 (17.5, 21.3)
Metabolic syndrome*^†^*					
Meeting 3 of 5 components	10.6 (9.5, 11.7)	10.2 (9.5, 10.9)	9.1 (8.5, 9.7)	8.0 (7.5, 8.5)	11.7 (11.2, 12.2)
Meeting 4 of 5 components	4.3 (3.6, 5.0)	3.5 (3.1, 3.9)	2.2 (1.9, 2.5)	2.0 (1.7, 2.3)	3.3 (3.0, 3.6)
Meeting 5 of 5 components	0.6 (0.3, 0.9)	0.4 (0.3, 0.5)	0.1 (0.0, 0.2)	0.1 (0.0, 0.2)	0.3 (0.2, 0.4)

	2017 N = 17 524	2018 N = 18 352	2019 N = 22 363	2020 N = 23 694	2021 N = 29 487
Total population	16.5 (16.0, 17.0)	13.7 (13.2, 14.2)	16.3 (15.8, 16.8)	20.6 (20.1, 21.1)	20.0 (19.5, 20.5)
Sex					
Male	22.2 (21.4, 23.0)	19.7 (18.9, 20.5)	24.0 (23.2, 24.8)	30.8 (30.0, 31.6)	30.3 (29.6, 31.0)
Female	9.3 (8.7, 9.9)	7.4 (6.9, 7.9)	8.0 (7.5, 8.5)	9.6 (9.1, 10.1)	8.8 (8.3, 9.3)
Age, years					
18-39	9.4 (8.7, 10.1)	13.1 (12.4, 13.8)	16.0 (15.4, 16.6)	20.3 (19.6, 21.0)	19.7 (19.1, 20.3)
40-59	18.8 (17.9, 19.7)	14.4 (13.5, 15.3)	16.6 (15.7, 17.5)	20.8 (19.9, 21.7)	19.9 (19.1, 20.7)
60-79	23.7 (22.3, 25.1)	14.3 (12.8, 15.8)	16.2 (14.8, 17.6)	21.2 (19.7, 22.7)	21.3 (19.8, 22.8)
≥80	18.6 (12.9, 24.3)	16.8 (11.1, 22.5)	17.1 (11.9, 22.3)	21.0 (15.9, 26.1)	19.8 (14.4, 25.2)
City					
Shanghai	14.9 (14.3, 15.5)	13.0 (12.4, 13.6)	17.3 (16.7, 17.9)	22.7 (22.0, 23.4)	21.6 (21.0, 22.2)
Nanjing	14.1 (12.2, 16.0)	13.2 (12.0, 14.4)	12.8 (11.6, 14.0)	13.7 (12.5, 14.9)	12.8 (11.7, 13.9)
Suzhou	22.3 (20.2, 24.4)	13.0 (11.3, 14.7)	13.7 (12.2, 15.2)	16.1 (14.6, 17.6)	15.6 (14.2, 17.0)
Nantong	22.1 (20.4, 23.8)	19.0 (17.3, 20.7)	16.5 (15.1, 17.9)	20.0 (18.5, 21.5)	22.1 (20.7, 23.5)
Metabolic syndrome*^†^*					
Meeting 3 of 5 components	12.0 (11.5, 12.5)	10.3 (9.9, 10.7)	11.6 (11.2, 12.0)	12.8 (12.4, 13.2)	12.8 (12.4, 13.2)
Meeting 4 of 5 components	4.1 (3.8, 4.4)	3.2 (2.9, 3.5)	4.0 (3.7, 4.3)	6.5 (6.2, 6.8)	6.0 (5.7, 6.3)
Meeting 5 of 5 components	0.5 (0.4, 0.6)	0.2 (0.1, 0.3)	0.6 (0.5, 0.7)	1.3 (1.2, 1.4)	1.1 (1.0, 1.2)

^*^Values are percentages (95% CI).

^*†*^Metabolic syndrome was defined according to the criteria of the Chinese Diabetes Society in 2013.

**Table 3.** Number of metabolic syndrome cases for different cities by sex in the study population, 2012-2021

		2012	2013	2014	2015	2016	2017	2018	2019	2020	2021
		N = 2859	N = 7724	N = 9810	N = 11 861	N = 14 600	N = 17 524	N = 18 352	N = 22 363	N = 23 694	N = 29 487
Shanghai	Participants	2421	4638	6349	7470	10 787	12 370	11 563	14 580	15 468	19 366
	Male	292	556	550	520	1102	1325	1090	1915	2716	3296
	Female	82	156	165	153	459	522	411	606	802	880
Nanjing	Participants	75	1599	1710	2259	947	1234	3264	3062	3033	3810
	Male	9	162	139	210	119	136	317	307	324	389
	Female	3	37	49	66	32	38	114	84	93	100
Suzhou	Participants	136	629	719	724	1141	1526	1460	2071	2322	2709
	Male	18	64	64	50	134	284	153	205	302	332
	Female	1	7	17	19	48	57	37	79	71	90
Nantong	Participants	227	858	1032	1408	1725	2394	2065	2650	2871	3602
	Male	30	98	108	145	283	436	296	337	445	609
	Female	7	15	23	42	52	94	96	101	130	188

### Decadal Trends in Adjusted Prevalence of MetS

**Figure 2.**
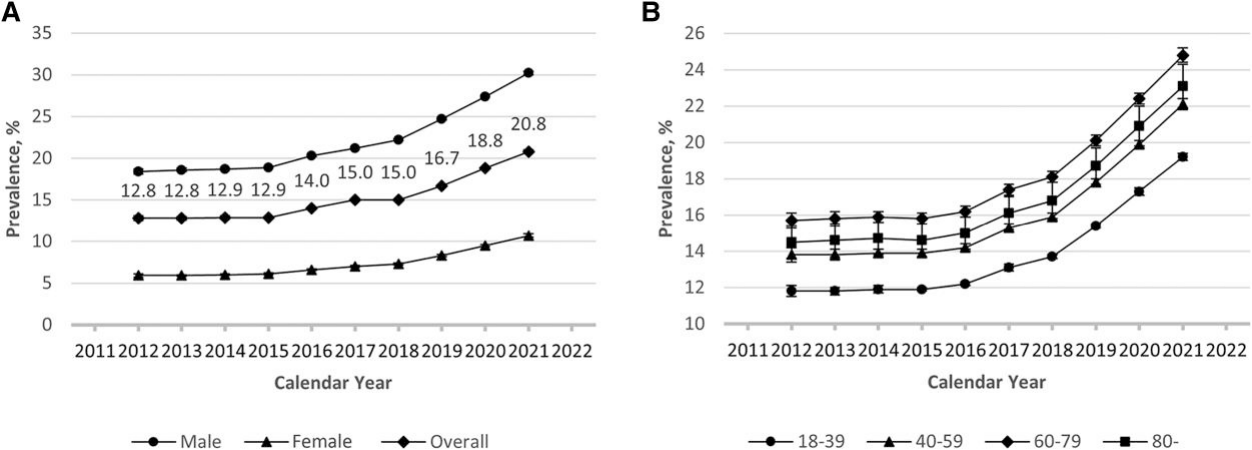
Adjusted prevalence of metabolic syndrome in the study population through 2012-2021 by gender (A) and by age group (B). The trends in the adjusted prevalence of MetS are described by restricted cubic splines and marked with percentage values for the overall prevalence.

**Figure 3.**
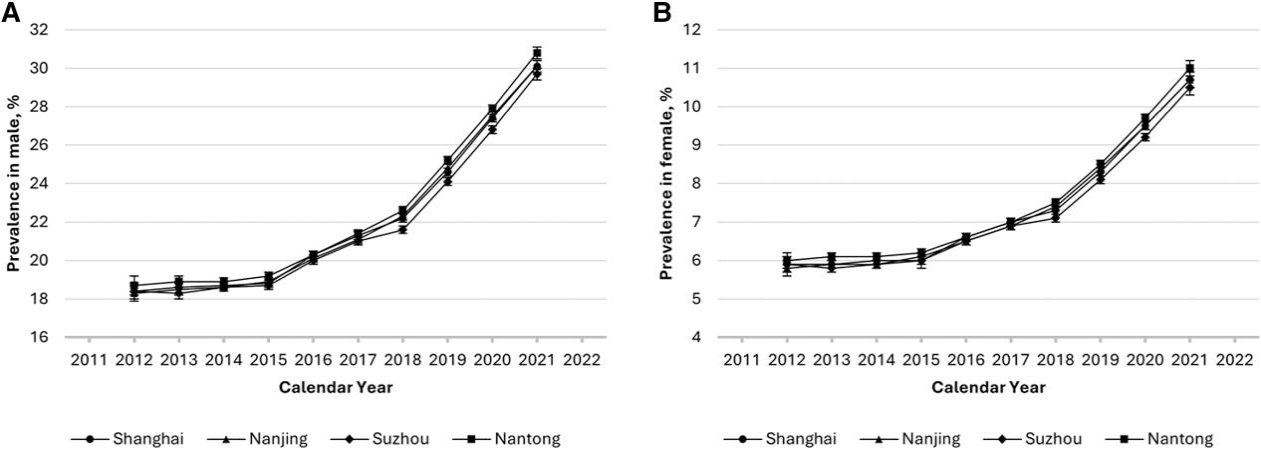
Adjusted prevalence of metabolic syndrome in the study population through 2012-2021 by cities in male (A) and in female (B). The trends in the adjusted prevalence of MetS are described by restricted cubic splines.

The adjusted trends were consistent with the crude prevalence results. There was a significant difference in the adjusted overall prevalence of MetS between 2012 and 2021 (*P* < .001). The prevalence of MetS adjusted for age was higher among men than women (Fig. 2A). Similarly, in all age groups, we found an initial increase in the adjusted prevalence through the early 2010s, followed by a rapid increase after 2016. The prevalence adjusted for sex was highest in the 60-79 age group, successively followed by those older than 80, then the 40-59 and 18-39 age groups (Fig. 2B). In the 4 cities, we observed a similar increasing pattern in the age-adjusted prevalence of MetS between 2012 and 2021 in male (Fig. 3A) as well as female individuals (Fig. 3B).

### Association Between Prevalence of MetS and Calendar Year and GDP Per Capita

**Figure 4.**
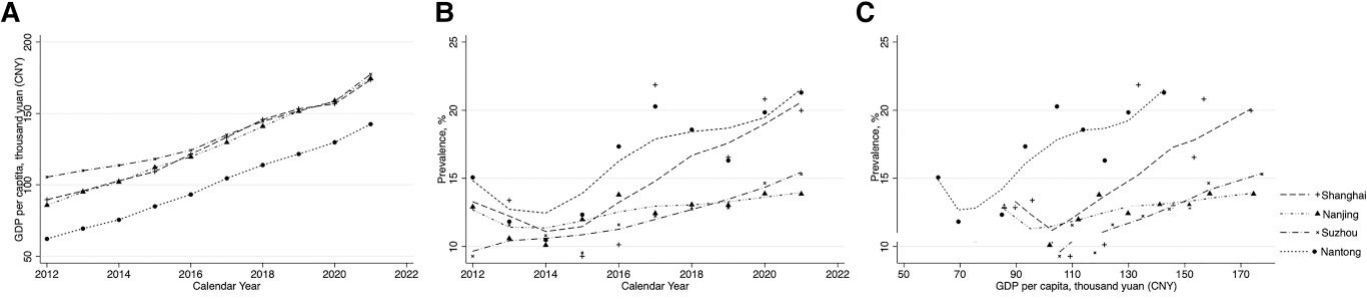
Sex and age-adjusted prevalence of metabolic syndrome in the study population through 2012-2021 by city. The prevalence of each city, each year was adjusted by age and sex using the overall distribution of age and sex in 2021 study samples. The data points of each city were connected using the LOWESS curve. (A) Gross domestic product (GDP) per capita over time in the 4 cities. (B) Sex and age-adjusted prevalence of MetS in 4 cities over time. (C) Sex and age-adjusted prevalence of MetS in 4 cities over GDP per capita.

From the LOWESS curve, we found that the GDP per capita of the 4 cities has increased at a smooth rate though 2012-2021. Among them, the GDP per capita of Shanghai, Nanjing, and Suzhou became very close after 2016, all higher than that of Nantong (Fig. 4A). The prevalence of MetS dropped though 2012-2014 in Suzhou and Nantong but increased rapidly after then. The increase of the prevalence in Shanghai started from 2016 and had the fastest growth rate. The prevalence in Nanjing had a general stabilization through the end of the study period (Fig. 4B). The trend in association between prevalence of MetS in the 4 cities and the GDP per capita was similar (Fig. 4C). In the multiple linear regression model, calendar year and GDP per capita were taken as independent variables, and sex and age-adjusted prevalence of MetS as the dependent variable. The results showed that sex and age-adjusted prevalence was positively correlated with calendar year (*P* < .001, 95% CI β: 0.648-1.954) and negatively correlated with GDP per capita (*P* = .030, 95% CI β: −0.136 to −0.007), suggesting that the prevalence rate of MetS decreased with the improvement of urban economic development level in the past decade.

## Discussion

This study was based on a large healthcare database estimating the trends in prevalence of MetS among urban residents in the most economically developed areas in China. We found a significant overall increase in MetS prevalence among health check-up adults, from 15.5% to 20.0%, from 2012 to 2021. This estimated pattern is consistent with the results in previous studies regarding the Chinese population [5, 16]. We observed considerable homogeneity among the study population across the decade in this study, estimating a reliable trend in prevalence. The previous studies reported a wide interval of prevalence in Chinese adults ranging from 13.3% to 46.3% [5], probably attributable to the heterogeneity of demographic and socioeconomic characteristics among their study populations as well as the different definitions of MetS. We used the most updated definition of MetS (CDS-2013) in our study because it is the most authoritative for diabetes and metabolic diseases in China.

Subgroup analysis showed that the adjusted prevalence among women increased slightly faster than in men through the past decade, from 5.9% to 10.7% in women vs from 18.4% to 30.2% in men, parallelly in all age strata. The fast-growing prevalence in young adults is important to note given that the health check-up clients are relatively young. Specifically, the health check-up population is a group of people dedicated to disease prevention in public health. Since MetS is more a risk factor for cardiovascular disease and diabetes rather than a syndrome comprising a set of symptoms, raising awareness of MetS in healthy asymptomatic populations may help identify patients who would benefit from improved screening and optimization of cardiovascular and diabetes risk profiles. Our study also found that the proportion of those who met 3 of 5 diagnostic components has increased from 10.6% to 12.8% through 2012-2021, whereas the number of those who met 5 of 5 diagnostic components in 2021 almost doubled from that in 2012. This may be due to the younger age of the disease onset, so that the results of mutual promotion of various factors between obesity, hypertension, hyperglycemia, and dyslipidemia have been presented. Our findings, which are based on more recent data, provide a valuable summary of the most recent prevalence of MetS, in contrast with prior publications reporting analyses. For instance, Liu et al reported that trends in the prevalence of populations aged 45 and above from 28 provinces decreased significantly between 2011 and 2015 by the CDS criteria [17]. A possible explanation for this decrease is that the middle-aged and elderly adults included came from regions with different economic development status, which may reflect lifestyle and education level among the study population.

The Yangtze River Delta region, where the 4 cities included in this study are located, represents the most economically developed areas in China. Another interest of this study is the correlation between the prevalence of MetS and city economic development levels. There exists some evidence of the association between the prevalence and T2DM. For example, Meo et al reported that the prevalence of T2DM was significantly associated with high GDP per capita in Arabic countries [12]. Similar results that higher GDP per capita seemed to have a higher prevalence of diabetes were reported in India [11]. However, the prevalence of MetS remains understudied. In the economic developed cases, although the high level of economic development is accompanied by higher energy intake and less physical activities, it also represents higher healthcare service input and accessibility. We are concerned about whether the prevalence rate still increases with the GDP per capita after controlling the variable factors of the calendar year. Our linear regression model revealed that calendar year had a positive effect (*P* < .001) on the prevalence of MetS, while GDP per capita had a negative effect (*P* = .030). This means that in the same calendar year, the prevalence of MetS demonstrates a negative association with GDP per capita, implying that economic development may strongly relate to changing in lifestyle and other risk factors for MetS. We put the calendar year and GDP per capita in the same linear regression model to account for the impact of GDP per capita growth year by year.

Our study also has several limitations. First, the selected cities are in the economically developed regions of China, and those individuals receiving annual health check in the commercial institutions may have relatively high levels of socioeconomic status. The population bias of this study cannot reflect the overall trend in prevalence of MetS in the general Chinese population. Second, MetS was defined by one-time physical and blood measurement results. Past medical history was collected based on self-report. Given that the purpose of this study is to explore the prevalence trend not the prevalence itself, using the same data collection method throughout the study years makes the data comparable. Third, we did not collect data on ethnicity, socioeconomic status, health care access, correlated health behaviors such as stress, unbalanced diet, physical inactivity, or alcohol and tobacco consumption, which are closely linked with MetS [18]. Although many risk factors have been documented in the literature to affect the prevalence of MetS, they are not available in the health check-up database.

## Conclusion

This study uses a health care database to investigate the trends in prevalence of MetS in the Chinese health check-up population. The overall and stratified prevalence of MetS significantly increased through 2012-2021, with an increasing trend among all age groups. Male and older age groups had higher prevalence of MetS. In the linear regression model of the prevalence of MetS, calendar year demonstrated a positive correlation with the prevalence of MetS, while GDP per capita had a negative correlation.

## Data Availability

Some or all datasets generated during and/or analyzed during the current study are not publicly available but are available from the corresponding author on reasonable request.

## Abbreviations

CDS: Chinese Diabetes Society
GDP: gross domestic product
HbA1c: glycated hemoglobin
HDL-C: high-density lipoprotein cholesterol
MetS: metabolic syndrome
T2DM: type 2 diabetes mellitus
